# Sugar-Sweetened Beverages and Symptom Complaints among School-Aged Children: A National Longitudinal Study

**DOI:** 10.3390/nu14030406

**Published:** 2022-01-18

**Authors:** Muqing Cao, Yanna Zhu, Yajun Chen, Jin Jing

**Affiliations:** Maternal and Child Health Department, School of Public Health, Sun Yat-sen University, Guangzhou 510080, China; caomq6@mail.sysu.edu.cn (M.C.); zhuyn3@mail.sysu.edu.cn (Y.Z.); chenyj68@mail.sysu.edu.cn (Y.C.)

**Keywords:** SSBs, symptom complaints, children, school-aged

## Abstract

The association between sugar-sweetened beverages (SSBs) and symptom complaints among school-aged children remains unclear. Children aged 6–17 years (*n* = 29,028) were recruited on the basis of a national school-based study. Data collection included two waves: the exposure was the amount and frequency of SSB consumption, collected in the 2013 autumn semester, and outcomes were defined as having clinical symptom complaints after two weeks of observation, collected in the 2014 spring semester. Symptom complaints were defined as fever, cough, headache, loose bowels, vomiting, sore throat, rash, conjunctival congestion, and stomachache. The associations between the amount/frequency of SSBs and symptoms complaints were explored by a general linear model, with adjustments made for socio-demographic and other lifestyle information. Among the 12,454 children (10.32 ± 3.15 years, 48.7% male) in the final analysis, the odds ratio for having symptom complaints (1.46, 95% CI: 1.10–1.95) among children whose SSB consumption was >75 mL/day was significantly higher than that among children who had no SSB consumption. Among children with a daily SSB intake of more than 75 mL, there was a higher risk for symptom complaints. We recommend more support for decreasing SSB consumption among children to minimize negative health outcomes.

## 1. Introduction

It has been suggested that the overconsumption of sugar-sweetened beverages (SSBs) is linked to a vast array of negative health outcomes in adulthood, including type 2 diabetes, cardiovascular disease, obesity, and dental caries [1]. SSBs may affect child health during a sensitive period [2]. Although our previous research showed a relationship between SSBs and metabolic index in childhood [3], chronic diseases are seldom present at such young age. According to a German study, clinical symptom complaints are common among school-aged children [4]. Another study indicated that symptoms such as medically unexplained abdominal pain and headache contributed to more than half of school absences [5], highly impacting academic performance [6]. It is unclear how these medically unexplained symptoms occurred, but diet advice may affect medically unsolved symptoms in childhood [7], indicating that these symptoms could be related to food.

SSBs consumption is related to inflammation, as large amounts of dietary sugars increase tumor necrosis factor alpha (TNF-a) levels [8], a higher cortisol release [9], and higher other inflammatory cytokine levels [10]. An intervention study among children who had nasal congestion symptoms showed that decreased SSB consumption was related to significantly reduced proinflammatory markers and increased anti-inflammatory markers, as well as symptom improvements [11], which indicated a possible mechanism by which SSBs are related to symptom complaints. On the other hand, high SSB consumption is correlated with mood disorders, such as a higher risk for depression [12], while mood disorders are also related to somatic symptom complaints [13,14,15]. Thus, mood disorders could be the potential mechanism of the SSBs–somatic symptom complaints association. We hypothesize that higher consumption of SSBs among children is associated with more symptom complaints, such as fever and abdominal pain, and we aim to confirm the association in a large sample size population-based study. Data from children aged 6–17 years who consumed SSBs were collected in the autumn semester, while symptom complaints were collected in the following spring semester via school classes. The amount and frequency of SSB consumption were explored by generalized linear models to clarify their potential relationship with symptom complaints. This study adds to the literature by studying SSBs and negative health outcomes among children, using data from a national survey in China. In addition, we established multilayer models; socio-demographic factors; lifestyle, including physical activity, sedentary time, sleep; and early life factors, including whether low birthweight and breastfeeding were adjusted step-by-step, in order to eliminate potential cofounders.

## 2. Methods

### 2.1. Participant Enrollment, Study Design, and Procedure

**Figure 1:**
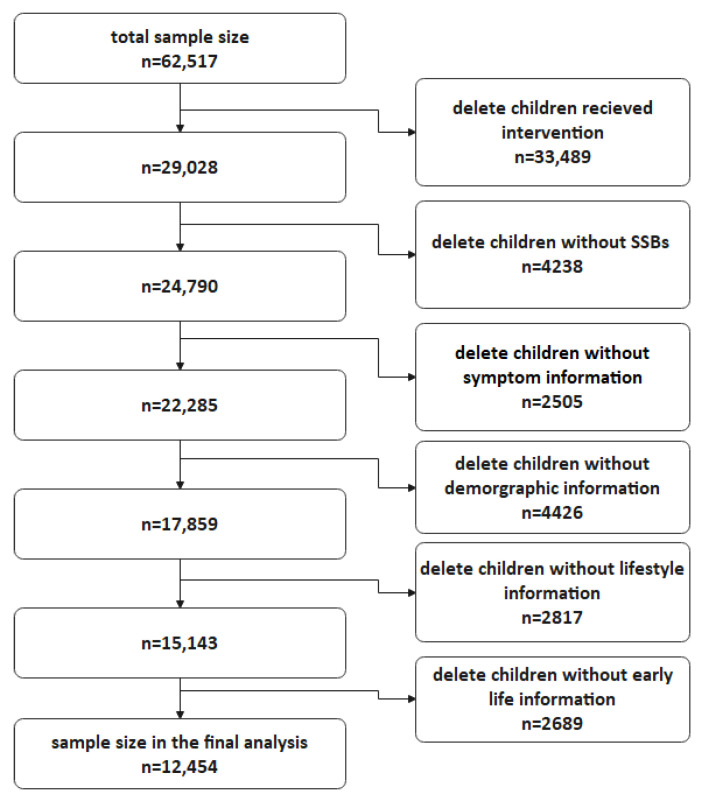
Flowchart of database cleaning.

All children in this study were derived from the control group without intervention in a school-based national intervention study, the protocol of which was published in 2015 [16]. Briefly, children were recruited from seven provinces/municipalities in China during September and November 2013, representing north (Tianjin), northeast (Liaoning), northwest (Ningxia), east (Shanghai), central (Changsha), southwest (Chongqing), and south (Guangzhou) China in terms of population geography. Multistage stratified cluster random sampling was conducted, and all children and their parents who agreed to join the study were asked to answer a questionnaire containing lifestyle information in the autumn semester of 2013 (September to November), as well as answer a questionnaire that retrospectively collected symptoms in the spring semester 2014. In total, there were 62,517 children aged 6–17 who agreed to participate in the intervention study, and the whole sample of this research came from 29,028 children in the control group. Children were excluded if they lacked data on sugar-sweetened beverage intake (SSBs, *n* = 4238), symptoms (*n* = 2505), socio-demographic information (*n* = 4426), physical activity/sedentary behavior or sleep duration (*n* = 2817), and breastfeeding/birthweight (*n* = 2689). There were 12,454 children included in the final analysis (Figure 1). This study was approved by the Sun Yat-sen University Ethics Committee, and all parents/guardians of the children signed the informed consent.

### 2.2. Questionnaire Survey

At the baseline stage, a standardized questionnaire that contained two parts was designed to collect children’s information. For the self-reported part, physical activity, sedentary behavior, sleep duration, and dietary intake information were collected by several questions. For the parent-reported part, child information, including demographic data and early life history (breastfeeding and if preterm), was collected by questions. Questionnaires were developed on the basis of the information, motivation, and behavioral skills model [17], which was piloted and revised in the early stages of the project to be feasible for children/parents. The questionnaires were assigned to students in their classes. Children would finish answering the self-reported questionnaire if they were 9 years old; otherwise, the researchers texted each parent to instruct them to help their children to fill in the questionnaire, finish the parent-reported questionnaire, and ensure that their children brought the two questionnaires back to school in a week. Well-trained researchers collected them from each class and implemented quality control methods. Details of the questionnaire are described elsewhere [18].

At the follow-up stage, a standardized self-reported child questionnaire was designed to collect symptoms retrospectively; the delivery and recollection procedure for the questionnaire was similar to that mentioned above.

### 2.3. Exposure: Sugar-Sweetened Beverage Intake Amount and Frequency

Sugar-sweetened beverage consumption was determined by asking the following questions: “In the last week, on how many days did you have a sugar-sweetened beverage? On those days, how many servings of sugar-sweetened beverages did you usually take each day? One serving of a sugar-sweetened beverage is 250 mL. A sugar-sweetened beverage includes juice with sugar, soda (e.g., Coca-Cola), milk drinks, energy drinks (e.g., Red Bull), and other beverages that contain sugar.” We listed the examples above because they were the most common and most popular sugar-sweetened beverages in the urban areas of China. Children should first specify the number of days that they had SSB intake in the past week, and then specify the amount of SSB intake each day.

SSB intake amounts were calculated and further grouped as “never have” [19], “small amount (no more than 75 mL per day)”, and “large amount (75 mL or more per day)”.

SSB intake frequency was calculated and further grouped as “never have”, “twice or less per week”, and “three or more times per week”.

### 2.4. Outcome: Symptoms

The symptoms were collected by a series of questions. Children were asked: “In the last two weeks, have you ever presented the symptoms below? 1. No symptoms at all, 2. Fever, 3. Cough, 4. Headache, 5. Loose bowels, 6. Vomiting, 7. Sore throat, 8. Rash, 9. Conjunctival congestion, 10. Stomachache, 11. Other symptoms, please specify.” Children were asked to choose “yes” or “no” for each symptom if they did not choose the first choice.

Symptoms were further grouped as “no reported” and “once or more reported”.

## 3. Covariates

### 3.1. Food Intake except for SSBs

Fruit intake was obtained by asking the following questions: “In the last week, on how many days did you have fruit intake? On those days, how many servings of fruit did you usually have each day? One serving of fruit is equivalent to the size of an adult’s fist”.

Vegetable intake was calculated by asking the following questions: “In the last week, on how many days did you have vegetable intake? On those days, how many servings of vegetables did you usually have each day? One serving of vegetables is equivalent to the size of an adult’s fist”.

Meat intake was obtained by asking the following questions: “In the last week, on how many days did you have meat intake? On those days, how many servings of meat did you usually take each day? A serving of meat is equivalent to the size of an adult’s palm”. If children felt unsure about the size of food, they were instructed to check the last page of the questionnaire, which showed a picture that was equivalent to one adult’s fist.

### 3.2. Moderate Physical Activity, Sedentary Behavior, and Sleep Duration

Moderate-intensity physical activity (MPA) was determined by asking the following questions: “In the last week, on how many days did you partake in moderate-intensity physical activity? On those days, how many hours of moderate-intensity physical activity did you undertake? Moderate-intensity physical activity means activities that cause mild sweating and slight exhaustion; for instance, bicycling, playing table tennis, and playing badminton, but walking is excluded”.

Sedentary behavior was assessed by the following question: “For how many hours do you sit or lie down at school and home (excluding sleeping) each week?”

Sleep duration was assessed by the following question: “For how many hours do you usually sleep each night? <7 h, 7–9 h, >9 h. Please choose the choice that is closest to your sleep duration”.

### 3.3. Early Life History

Birth weight was determined by asking parents “How many jins (1/2 kg) and liangs (50 g) did your child weigh at birth?” Parents were required to specify the weight that their children were at birth. Birth weight was converted to grams and categorized as low birth weight (<2500 g), normal birth weight (≥2500 g and <4000 g), or macrosomia (≥4000 g).

Breastfeeding history was collected by asking parents the following questions: “Has your child been breastfed? If so, please specify the age when breastfeeding stopped”. We further grouped breastfeeding history as “<6 months or none, ≥6 months”.

### 3.4. Statistical Analysis

We used SPSS 25.0 for data analysis. Descriptive statistics were calculated for all variables, including continuous (presented as mean values ± standard errors) and categorical variables (presented as proportions). Chi-squared tests were used to evaluate the differences in categorical variables, while *t*-tests, one-way ANOVA (normal distribution) or Mann–Whitney *U* test, and Kruskal–Wallis test (abnormal distribution) were used to evaluate the differences in continuous variables. Multivariate logistic regression was used in four models, and the odds ratios and the corresponding 95% CIs were presented. We first presented a crude model with no confounding variables adjusted, and then child age, gender, being an only child, household income, residence, research area, ethnic background, and maternal education level were introduced into adjusted model 1; then, we introduced moderate-intensity physical activity time, sedentary lifestyle time, sleep duration, low birth weight, and breastfeeding for longer than 6 months in adjusted model 2. Finally, sugar-sweetened beverage intake frequency/amount was introduced in adjusted model 3, depending on the outcome. *p*-values less than 0.05 were considered statistically significant.

Sensitivity analyses on the main outcomes (association between SSB intake amount/frequency and symptoms) were performed in participants of Han ethnic background, and similar results were found (attached in the Supplemental Material, Table S1).

## 4. Results

There were 12,454 children in the final analysis, of whom 34.9% reported symptoms at least once in the last semester. The mean age was 10.32 years (standard deviation (SD) 3.15 years), with 48.7% being boys. The majority of the children were of a Han ethnic background (95.4%) from urban areas (60.4%). Half of the children had a mother with an education level of 9 years or less (50%). However, only 10% of children came from a family with a monthly income of more than CNY 12,000 (approximately USD 1800). The most common moderate physical activity time was 0.75 h or less per day (34.7%), and 41.2% of the children had 6.5 h or more of sedentary time daily. As for sleep time, 7–9 h was the most commonly reported (59.5%). As for sugar-sweetened beverage intake, the average intake amount was 1.59 ± 1.76 serves/week, and the average intake frequency was 2.74 ± 5.07 times/week. The most common report was a small amount (no more than 75 mL per day, 39.1%) and twice or less per week (44.9%). Most children had normal birth weight (90.2%) and the minority of children (37.1%) were not breastfed for longer than 6 months.

Compared with the no-symptoms group, children with symptoms were older (10.44 ± 3.18 vs. 10.26 ± 3.14; *p* = 0.002), were more commonly girls (37.8 vs. 31.8%, girls vs. boys; *p* < 0.001), from a minority ethnic background (39.7 vs. 34.7%; *p* = 0.013), with less educated mothers (9 years or below 37.5%, 9–12 years 33.0,%, 13–15 years 31.2%, 16 years or above 32.1%), had less household income per month (CNY 5000 or below 37.4%, CNY 5001–12000 33.4%, CYN 12,001 or above 32.3%; *p* < 0.001), and lived in a rural area (38.5 vs. 32.5%; *p* < 0.001). Children with symptoms also had less sleep (<7 h 39.5%, 7–9 h 34.1%, 9 h or more 34.4%; *p* < 0.001), a greater SSB intake amount (large amount 38.9%, small amount 34.4%, never 32.5%; *p* < 0.001), and a greater SSB intake frequency (three times or more per week 38.1%, twice or less 35.3%, never 32.5%; *p* < 0.001); however, less of them were breastfed for > 6 months (33.6 vs. 35.6%, *p* = 0.024). Basic characteristics of research population according to whether symptoms were presented or not are presented in Table 1.

**Table 1:** Basic characteristics of research population according to whether symptoms were presented or not.

Characteristics		Overall (*n* = 12,454)	Symptom Presence		*p*-Value
			Yes (*n* = 4345)	No (*n* = 8109)	
Age (year)		10.32 (3.15)	10.44 (3.18)	10.26 (3.14)	0.002
Child gender (%)	Boys	6059 (48.7)	1929 (31.8)	4130 (68.2)	<0.001
	Girls	6395 (51.3)	2416 (37.8)	3979 (62.2)	
Ethnic background (%)	Han	11,880 (95.4)	4117 (34.7)	7763 (65.3)	0.013
	Minority	574 (4.6)	228 (39.7)	346 (60.3)	
Maternal education level (%)	9 years or below	6227 (50.0)	2334 (37.5)	3893 (62.5)	<0.001
	9–12 years	3219 (25.8)	1061 (33.0)	2158 (67.0)	
	13–15 years	1705 (13.7)	532 (31.2)	1173 (68.8)	
	16 years or above	1303 (10.5)	418 (32.1)	885 (67.9)	
Residence (%)	Urban	7525 (60.4)	2449 (32.5)	5076 (67.5)	<0.001
	Rural	4929 (39.6)	1896 (38.5)	3033 (61.5)	
Household income per month (%)	CNY 5000 or below	4014 (32.2)	1501 (37.4)	2513 (62.6)	<0.001
	CNY 5001–12,000	3756 (30.2)	1253 (33.4)	2503 (66.6)	
	CNY 12,001 or above	1246 (10.0)	402 (32.3)	844 (67.7)	
	Refused to answer	3438 (27.6)	1189 (34.6)	2249 (65.4)	
Research area (%)	Eastern and southern coast	4289 (34.4)	1267 (29.5)	3022 (70.5)	<0.001
	Northern	4530 (36.4)	1694 (37.4)	2836 (62.6)	
	Middle and western	3635 (29.2)	1384 (38.1)	2251 (61.9)	
Daily moderate physical activity time (%)	0.75 h or less	4320 (34.7)	1523 (34.3)	2797 (65.7)	0.603
	0.75–3 h	4234 (34.0)	1452 (34.3)	2782 (65.7)	
	3 h or more	3900 (31.3)	1370 (35.1)	2530 (64.9)	
Daily sedentary lifestyle time (%)	2.5 h or less	3340 (26.8)	1186 (35.5)	2154 (64.5)	0.634
	2.5–6.5 h	3989 (32.0)	1391 (34.9)	2598 (65.1)	
	6.5 h or more	5125 (41.2)	1768 (34.5)	3357 (65.5)	
Daily sleep time (%)	7 h or less	3423 (27.5)	1178 (39.5)	2245 (60.5)	<0.001
	7–9 h	7406 (59.5)	2525 (34.1)	4881 (65.9)	
	9 h or more	1625 (13.0)	642 (34.4)	983 (65.6)	
Consumption of vegetables (%)	0–6 serves/week	2511 (20.3)	892 (35.5)	1619 (64.5)	0.211
	7–14 serves/week	7228 (58.4)	2478 (34.3)	4750 (65.7)	
	15 or more serves/week	2634 (21.3)	949 (36.0)	1685 (64.0)	
Consumption of fruits (%)	0–5 serves/week	4012 (32.5)	1400 (34.9)	2612 (65.1)	0.577
	6–11 serves/week	4913 (39.8)	1735 (35.3)	3178 (64.7)	
	12 or more serves/week	3418 (27.7)	1169 (34.2)	2249 (65.8)	
Consumption of meats (%)	0–4 serves/week	4437 (36.0)	1586 (35.7)	1636 (34.2)	0.314
	5–7 serves/week	4779 (38.8)	1636 (34.2)	3143 (65.8)	
	8 or more serves/week	3113 (25.2)	1090 (35.0)	2023 (65.0)	
Sugar-sweetened beverage intake amount (SSBsM, %)	Never	4385 (35.2)	1425 (32.5)	2960 (67.5)	<0.001
	Small amount (no more than 75 mL per day)	4873 (39.1)	1677 (34.4)	3196 (65.6)	
	Large amount (75 mL or more per day)	3196 (25.7)	1243 (38.9)	1953 (61.1)	
Sugar-sweetened beverage intake frequency (SSBsF, %)	Never	4385 (35.2)	1425 (32.5)	2960 (67.5)	<0.001
	Twice or less per week	5594 (44.9)	1976 (35.3)	3618 (64.7)	
	Three times or more per week	2475 (19.9)	944 (38.1)	1531 (61.9)	
Birth weight (%)	Normal	11,229 (90.2)	3896 (34.7)	7333 (65.3)	0.196
	Low birthweight	374 (3.0)	128 (34.2)	246 (65.8)	
	Macrosomia	851 (6.8)	321 (37.7)	530 (62.3)	
Breast feeding less than 6 months (%)	Yes	4615 (37.1)	1552 (33.6)	3063 (66.4)	0.024
	No	7839 (62.9)	3063 (35.6)	5046 (64.4)	

Children with a large amount SSB intake had older age (11.4 ± 3.32, 9.99 ± 3.03, and 9.91 ± 2.98 for large amount, small amount, and never, respectively; *p* < 0.001), were mostly boys (55.3, 47.7, and 44.9% for large amount, small amount, and never, respectively; *p* < 0.001), were from rural areas (42.6, 37.9, and 39.2% for large amount, small amount, and never, respectively; *p* < 0.001), had less educated mothers (9 years or below vs. 16 years or above: 55.0 vs. 8.1, 48.1 vs. 10.5, and 48.5 vs. 12.1% for large amount, small amount, and never, respectively; *p* < 0.001), and were from middle and western areas of China (31.1, 28.8, and 28.2% for large amount, small amount, and never, respectively; *p* < 0.001) relative to children with small SSB intakes or children who never drank SSBs. Large SSB intake was associated with longer daily physical activity time (3 h or more: 34.0, 31.2, and 29.5% for large amount, small amount, and never, respectively; *p* < 0.001) and sedentary time (6.6 h or more: 45.8, 39.1, and 40.0% for large amount, small amount, and never, respectively; *p* < 0.001); however, they were more likely to report <7 h of sleep time (19.6, 11.1, and 10.4% for large amount, small amount, and never, respectively; *p* < 0.001), less consumption of vegetables (15 or more serves per week: 21.6, 19.2, and 23.4% for large amount, small amount, and never, respectively; *p* < 0.001) but more meats (8 or more servings/week: 34.2, 22.1, and 22.2% for large amount, small amount, and never, respectively; *p* < 0.001), had a greater proportion of macrosomia (8.4%, 6.6%, and 5.9% for large amount, small amount, and never, respectively; *p* < 0.001), and were more likely to report symptoms (38.9, 34.4, and 32.5% for large amount, small amount, and never, respectively; *p* < 0.001). Basic characteristics of research population according to sugar-sweetened beverage amount are presented in Table 2.

**Table 2:** Basic characteristics of research population according to sugar-sweetened beverage amount.

Characteristics		Never (*n* = 4385)	Small Amount (*n* = 4873)	Large Amount (*n* = 3196)	*p*-Value
Age (year)		9.91 (2.98)	9.99 (3.03)	11.40 (3.32)	<0.001
Child gender (%)	Boys	1967 (44.9)	2324 (47.7)	1768 (55.3)	<0.001
	girls	2418 (55.1)	2549 (52.3)	1428 (44.7)	
Ethnic background (%)	Han	4175 (95.2)	4663 (95.7)	3042 (95.2)	0.441
	Minority	210 (4.8)	210 (4.3)	154 (4.8)	
Maternal education level (%)	9 years or below	2126 (48.5)	2344 (48.1)	1757 (55.0)	<0.001
	9–12 years	1070 (24.4)	1320 (27.1)	829 (25.9)	
	13–15 years	658 (15.0)	695 (14.3)	352 (11.0)	
	16 years or above	531 (12.1)	514 (10.5)	258 (8.1)	
Residence (%)	Urban	2666 (60.8)	3042 (62.1)	1835 (57.4)	<0.001
	Rural	1719 (39.2)	1849 (37.9)	1361 (42.6)	
Household income per month (%)	CNY 5000 or below CNY 5001–12,000	1445 (33.0)	1502 (30.8)	1067 (33.4)	0.082
		1275 (29.1)	1523 (31.3)	958 (30.0)	
	CNY 12,001 or above Refused to answer	435 (9.9)	509 (10.4)	302 (9.4)	
		1230 (28.1)	1339 (27.5)	869 (7.0)	
Research area (%)	Easter and southern coast	1620 (36.9)	1678 (34.4)	991 (31.0)	<0.001
	Northern	1530 (34.9)	1790 (36.7)	1210 (37.9)	
	Middle and western	1235 (28.2)	1405 (28.8)	995 (31.1)	
Daily physical activity time (%)	0.75 h or less	1700 (38.8)	1585 (32.5)	1035 (32.4)	<0.001
	0.75–3 h	1391 (31.7)	1769 (36.3)	1074 (33.6)	
	3 h or more	1294 (29.5)	1519 (31.2)	1087 (34.0)	
Daily sedentary lifestyle time (%)	2.5 h or less	1185 (27.0)	1354 (27.8)	801 (25.1)	<0.001
	2.5–6.5 h	1447 (33.0)	1612 (33.1)	930 (29.1)	
	6.5 h or more	1753 (40.0)	1907 (39.1)	1465 (45.8)	
Daily sleep time (%)	9 h or more	1349 (30.8)	1429 (29.3)	645 (20.2)	<0.001
	7–9 h	2579 (58.8)	2902 (59.6)	1925 (60.2)	
	7 h or less	457 (10.4)	542 (11.1)	626 (19.6)	
Consumption of vegetables (%)	0–6 serves/week	700 (16.1)	1032 (21.3)	779 (24.5)	<0.001
	7–14 serves/week	2631 (60.5)	2884 (59.5)	1713 (53.9)	
	15 or more serves/week	1016 (23.4)	932 (19.2)	686 (21.6)	
Consumption of fruits (%)	0–5 serves/week	1341 (30.8)	1642 (34.0)	1029 (32.5)	0.001
	6–11 serves/week	1797 (41.3)	1908 (39.6)	1208 (38.1)	
	12 or more serves/week	1211 (27.8)	1274 (26.4)	933 (29.4)	
Consumption of meats (%)	0–4 serves/week	1664 (38.4)	1855 (38.4)	918 (29.0)	<0.001
	5–7 serves/week	1708 (39.4)	1906 (39.5)	1165 (36.8)	
	8 or more serves/week	964 (22.2)	1067 (22.1)	1082 (34.2)	
Sugar-sweetened beverage intake frequency (SSBsF, %)	Never	4385 (100)	0 (0)	0 (0)	<0.001
	Twice or less per week	0 (0)	4776 (98.0)	818 (25.6)	
	Three times or more per week	0 (0)	97 (2.0)	2378 (74.4)	
Birth weight (%)	Normal	3980 (90.8)	4408 (90.5)	2841 (88.9)	<0.001
	Low birthweight	148 (3.4)	141 (2.9)	85 (2.7)	
	Macrosomia	257 (5.9)	324 (6.6)	270 (8.4)	
Breast feeding less than 6 months (%)	Yes	1633 (37.2)	1834 (37.6)	1148 (35.9)	0.281
	No	2752 (62.8)	3039 (62.4)	2048 (64.1)	
Symptom reported (%)	Yes	1425 (32.5)	1677 (34.4)	1243 (38.9)	<0.001
	No	2960 (67.5)	3196 (65.6)	1953 (61.1)	

Adjusting for age, gender, and other socio-demographic factors in the analysis, we found that the likelihood of symptoms being reported was higher among children reporting SSB intake (large amount: odds ratio (OR) 1.33, 95% confidence interval (CI) 1.20–1.47; small amount: OR 1.09, 95% CI 1.00–1.19; Table 3, adjusted model 1), compared with children who never drank SSBs. Children reporting SSB intake were more likely to report symptoms when we adjusted for moderate physical activity, sedentary lifestyle time, sleep duration, low birth weight, and breastfeeding for longer than 6 months (large amount: OR 1.32, 95% CI 1.20–1.46; small amount: OR 1.09, 95% CI 1.00–1.19; Table 3, adjusted model 2).

More frequent SSB intake was associated with an increased likelihood of symptom reports (three times or more per week: OR 1.28, 95% CI 1.16–1.43; twice or less per week: OR 1.12, 95% CI 1.04–1.23; compared with never; Table 3, adjusted model 2). 

## 5. Discussion

This study aimed to gain an understanding of the relationship between potential negative health outcomes and SSB consumption among a school-aged child population in China by examining the association of amount and frequency of SSB consumption with clinical symptom complaints between the ages of 6 and 17 years. Using longitudinal data from a national multicenter study, we observed that >75 mL/day consumption of SSBs was associated with an increased likelihood of clinical symptom complaints six months later. Children who consumed SSBs three or more times per week also showed more symptom complaints, but this effect may be modified by the amount of SSBs consumed.

Several potential mechanisms could explain this relationship. Depending on whether clinical symptom complaints were medically explained (such as infection) or unexplained (functional, which could not be explained by any physical diseases), we explain these associations separately. Referring to clinical symptoms explained by physically related disease, the large sugar content in SSBs provides a higher glycemic index (GI) compared with other liquids [20]. An animal model showed that high glucose levels exacerbate inflammatory effects through the activation of cytokines, such as transforming growth factor (TGF) and the promotion of Th17 cell differentiation [10] and dysregulated immunity homeostasis [21]. Even in terms of the most frequently mentioned chronic diseases in relation to SSBs, obesity and systematic inflammation have been widely observed [22]. Moreover, SSB-related hyperglycemia is associated with infection and its clinically related symptoms [23]. As fever, headache, abdominal pain, etc., were also symptoms [24], SSB consumption may affect symptoms by inducing infection or chronic systemic inflammation; however, this speculation requires confirmation.

Medically unexplained symptom complaints are usually related to particular personality traits, such as nervousness and anxiety [25], as well as negative psychological suggestions [25]; these traits are also found among individuals who have a preference for sugar [26]. Moreover, addictive behavior is also frequently mentioned in SSB consumption, as sugar itself is believed to be addictive [27], and those who consume it share the same personality traits with individuals who frequently endure somatic complaints [28].

Food additives in SSBs are another aspect concerning children’s mental health, as artificial food colorings and preservatives are reported as being ADHD symptom risk factors [29], while caffeine and nonnutritive sweeteners are related to behavioral and mental problems [30]. Although there is no evidence that food additives increase the risk of symptom complaints, their potential effects on endocrine system disruption, obesogenic activity, oxidative stress, and immunosuppression [31] are of great concern and may have an unpredictable impact, especially if exposure occurs during childhood.

We used the “never have” group as the reference in our analysis because, according to Dietary Guidelines for Chinese School-age Children—2016, 3rd edition [19], SSBs are not recommended for school-aged children at all. However, SSB consumption among children has increased dramatically in recent decades. According to data from the 2010s, children from the United States had 155 kcal intake from SSBs daily [32], and 6–17-year-old children in China had an average SSBs consumption of 181 g/day and 2.2 times per week in 2020 [33]. Only 35.2% of children in our study reported no SSB consumption in the last week, which is lower than the data of 2020 [33], confirming the increasing trend of SSBs in China. In the last 10 years, only data from the North American area reported a decrease in SSBs [34], while data from eastern China 2019 showed that about 40% of high school children consumed SSBs once per week [35]. Thus, more efforts regarding the promotion of healthy diets are needed in China to decrease negative SSB-related outcomes.

There are a few limitations to this study that should be addressed. First, we did not distinguish between medically and non-medically explained symptoms; thus, a stratified analysis could not be performed. In addition, a relatively high proportion of children did not report either SSB consumption or symptom complaints, and this may have caused information bias. Sugar-sweetened beverage collection did not exclude nonnutritive sweeteners, which compromised a more solid conclusion regarding sugar and the relationship with symptom complaints. We did not include the consumption of other sweet products; thus, we cannot address the effects of total sugar consumption on symptom complaints. Body mass index or other indicators that could represent weight status were not included in the study; thus, we could not know whether the association between SSBs and symptoms are affected by body weight. The data collection was performed in 2013–2014, and therefore only represents a situation from 7 years ago. Data regarding SSB consumption patterns were collected 6 months before data regarding symptom complaints, and the consumption patterns could have changed during the interim period. The season change was not considered in the study design, and some winter–spring epidemic diseases such as flu may cause fever, cough, and other symptoms we observed, thus biasing the results.

In conclusion, school-aged children who had excessive SSB consumption were found to have a higher likelihood to report symptom complaints six months later. This study is the first to explore the relationship between SSBs and clinical symptoms and adds value to the field of SSBs and negative health outcomes. The research results indicate that SSBs are not only a risk factor for diagnosed diseases, such as obesity, diabetes, and ADHD, but are also potentially harmful to the daily well-being of children. These results need more longitudinal and interventional studies to confirm their validity. Efforts to decrease SSB consumption are necessary to promote health and well-being among school-aged children in China.

## Figures and Tables

**Table 3:** Odds ratio and 95% confidence interval of symptom prevalence among different sugar-sweetened beverage intakes.

		Crude Model ^a^	Adjusted Model 1 ^b^	Adjusted Model 2 ^c^
Sugar-sweetened beverage intake amount	Never	Reference	Reference	Reference
	Small amount	1.09 (1.00–1.19)	1.09 (1.00–1.19) *	1.09 (1.00–1.19) *
	Large amount	1.32 (1.20–1.45) *	1.33 (1.20–1.47) *	1.32 (1.20–1.46) *
Sugar-sweetened beverage intake frequency	Never	Reference	Reference	Reference
	Twice or less per week	1.13 (1.04–1.23) *	1.14 (1.04–1.24) *	1.13 (1.04–1.23) *
	Three times or more per week	1.28 (1.16–1.42) *	1.29 (1.17–1.44) *	1.28 (1.16–1.43) *

* *p* < 0.05. ^a^ Crude model had no adjustment. ^b^ Adjusted child age, gender, one child, household income, residence, research area, ethnic background, and maternal education level. ^c^ Adjusted variants in adjusted model 1 plus food intake, moderate physical activity time, sedentary lifestyle time, sleep duration, low birth weight, and breastfeeding for longer than 6 months.

## Data Availability

The data presented in this study are available on request from the corresponding author. The data are not publicly available due to ethical restrictions.

## Supplementary Materials

The following supporting information can be downloaded at: https://www.mdpi.com/article/10.3390/nu14030406/s1, Table S1: Odds ratio and 95% confidence interval of inflammation-related symptom prevalence among different sugar-sweet beverage intake (only included Han ethnic background children, *n* = 11,707).
