# Supplementary material for: Sugar-Sweetened Beverages and Symptom Complaints among School-Aged Children: A National Longitudinal Study
Source: Nutrients. 2022 Jan 18;14(3):406. doi: 10.3390/nu14030406 (PMC8839686; doi:10.3390/nu14030406)
Supplement: Supplementary file 1 [file nutrients-14-00406-s001.zip › nutrients-1542342-supplementary.pdf]

Table S1. Odds ratio and 95% confidence interval of inflammation-related symptom prevalence among different sugar-sweet beverage intake. (only included Han ethnic background children, n=11707) [a].

	Never		Small mount		Large mount	
Sugar sweet beverage intake mount	Referent	n=4107	1.20 (0. 99-1.44)	n=4597	1.57 (1.17-2.10)	n=3033
	Never		Twice or less per week		Three or more times per week	
Sugar sweet beverage intake Frequency	Referent	n=4175	0.83 (0.69-1.01)	n=5350	0.77 (0.56-1.04)	n=2355

[a] adjusted child age, gender, one child, household income, residence, research area, maternal education level, moderate physical activity time, sedentary lifestyle time, sleep duration, low birth weight, and if breastfeeding longer than 6 months.
